# Abalone Viral Ganglioneuritis

**DOI:** 10.3390/pathogens9090720

**Published:** 2020-09-01

**Authors:** Serge Corbeil

**Affiliations:** CSIRO, Australian Centre for Disease Preparedness, 5 Portarlington Rd, East Geelong, VIC 3219, Australia; s.corbeil@csiro.au

**Keywords:** abalone viral ganglioneuritis, AVG, abalone herpesvirus, AbHV, Haliotid herpesvirus-1, HaHV-1, abalone, *Haliotis* spp.

## Abstract

Abalone viral ganglioneuritis (AVG), caused by Haliotid herpesvirus-1 (HaHV-1; previously called abalone herpesvirus), is a disease that has been responsible for extensive mortalities in wild and farmed abalone and has caused significant economic losses in Asia and Australia since outbreaks occurred in the early 2000s. Researchers from Taiwan, China, and Australia have conducted numerous studies encompassing HaHV-1 genome sequencing, development of molecular diagnostic tests, and evaluation of the susceptibility of various abalone species to AVG as well as studies of gene expression in abalone upon virus infection. This review presents a timeline of the most significant research findings on AVG and HaHV-1 as well as potential future research avenues to further understand this disease in order to develop better management strategies.

## 1. Introduction

Until recently, most studies on herpesviruses have been carried out on mammals, including humans, as research funding has mainly been aimed at solving problems of medical or agricultural nature. Therefore, knowledge of aquatic herpesviruses and their interactions with their hosts has remained sparse. This situation has changed since the early 1990s with the occurrence of ostreid herpesvirus-1 (OsHV-1), which impacted Pacific oyster (*Crassostrea gigas*) culture in countries such as France, USA, UK, New Zealand, and Australia [1,2,3,4,5]. The first herpesvirus infection of abalone, caused by the abalone herpesvirus (AbHV), was officially reported in 2003 in Taiwan [6], causing acute disease with rapid onset and high mortality of farmed *Haliotis diversicolor supertexta*. To this day (2020), Taiwanese farmed abalone still suffer from AVG, putting financial hardship on the industry [personal communication].

Although unknown at the time, AbHV is also believed to have caused significant economic damage to *H. diversicolor supertexta* cultured in Dongshan County of Fujian Province, China, in the late 1990s and early 2000s [7,8]. Following the severe impact of AVG in China, the Chinese abalone industry has moved away from growing the susceptible species *H. diversicolor supertexta* and has since reported few cases of AVG [personal communication]. However, an 11-year epidemiological study (2002 to 2013) [9] carried out on *H. diversicolor supertexta* and *H. discus hannai* grown along the coast of the South China Sea showed that AbHV infection was commonly found in otherwise healthy abalone. However, in one site under investigation, AVG mortalities were observed only in *H. diversicolor supertexta*. The authors concluded that either the environmental conditions may have been unfavorable for disease development in *H. discus hannai* or that their natural immunity prevented outbreak of disease [9,10].

**Figure 1:**
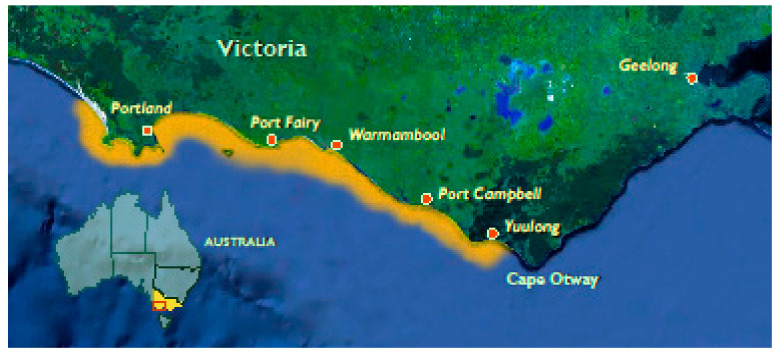
Range of abalone viral ganglioneuritis spread in 2010 Victoria, Australia (highlighted in orange).

In 2005–2006, outbreaks in both farmed and wild abalone populations along the coast of Victoria, Australia (Figure 1), were associated with the rapid onset of high mortalities (up to 90%) in all age classes of *H. rubra*, *H. laevigata*, and their hybrids [11]. Subsequently, several genetic variants of AbHV were identified in processing plants in Tasmania, the world’s largest producer of wild-caught abalone [12,13]. Since then, Australia has maintained a regular surveillance program (prior to interstate translocation and international shipping) and has not reported a case of AVG since 2011. Sequencing and phylogenetic analysis of AbHV revealed a high percentage of sequence homology with the OsHV-1 affecting bivalves [14]. Based on this phylogenetic sequencing work, AbHV was renamed Haliotid herpesvirus-1 (HaHV-1) and classified as a new species in a newly created genus *Aurivirus*, which, together with the genus *Ostreavirus,* comprises the family *Malacoherpesviridae* within the order *Herpesvirales* [15]. The risk that AVG still poses on abalone aquaculture is worrisome as world production has more than quadrupled between 2007 and 2017, increasing from 41,375 metric tons to 174,633 metric tons per year [16]. The abalone wild catch is more modest with a production of 6341 metric tons per annum in 2017 but remains a significant contributor to the local economies of some countries such as Australia, with wild abalone fisheries valued at AU$200m in 2017–2018 [16,17,18].

## 2. Clinical Signs and Pathology

**Figure 2:**
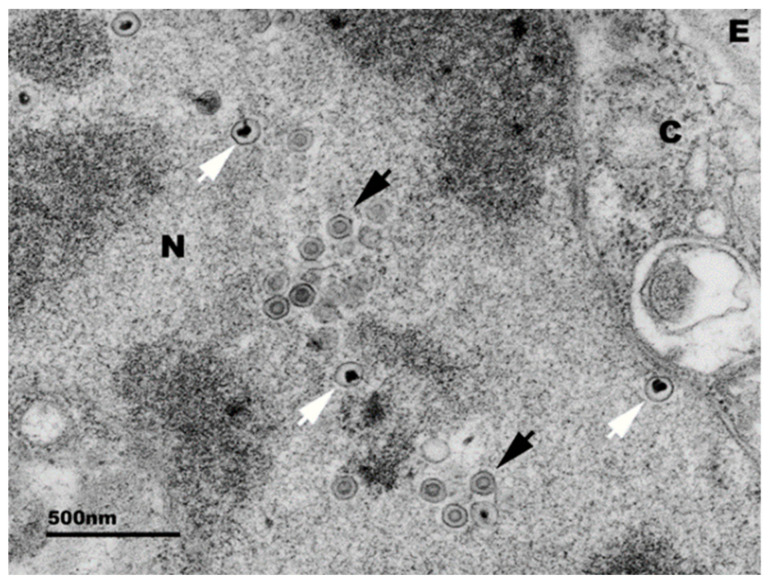
Electron micrograph of abalone pleuropedal ganglion cell infected with the Victorian HaHV-1 isolate. Black arrows = mature capsids, white arrow = immature capsids, N = nucleus, C = cytoplasm, E = extracellular space. Micrograph provided by Alex Hyatt and Sandy Cramery, CSIRO.

**Figure 3:**
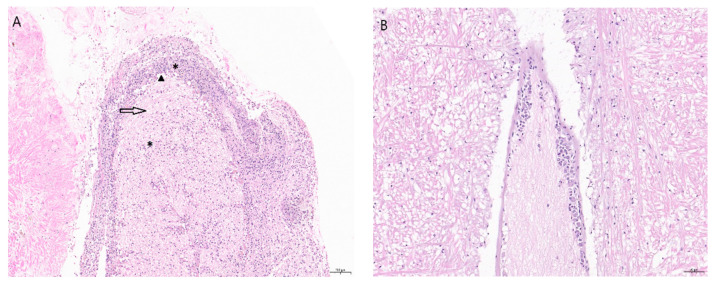
(**A**) Section of an abalone nerve cord and ganglion with abalone viral ganglioneuritis induced lesions. There is disruption to the normal morphology of the nerve, as seen by a marked hemocyte infiltrate (*), edema (arrow), and neuronal necrosis (arrowhead/black triangle) (hematoxylin and eosin staining). Magnification 10×. Scale bar 100 µm. (**B**) Normal ganglion. Magnification 20×. Scale bar 50 µm.

HaHV-1 is a spherical virus with an icosahedral core and an envelope of approximately 100 nm in diameter [19,20] (Figure 2). Infection of abalone is characterized by acute mortality and necrotizing ganglioneuritis [6,11]; therefore, the disease was named abalone viral ganglioneuritis. In *H. diversicolor supertexta*, lesions include necrosis of the cerebral ganglia and nerve bundles in the muscle of the foot as well as in the muscular layers beneath the visceral organs with associated hemocyte infiltrates [6]. Observation of prominent neurological lesions were originally performed through light and electron microscopy methods [6,11] (Figure 3).

Some clinical signs of infection differ between Australian and Taiwanese abalone in that moribund Australian abalone had a swollen mouth and prolapsed odontophore [11], while clinical signs of infection in Taiwanese abalone included mantle recession and muscle atrophy [6] but lacked the oral lesions observed in Australia. Chen et al. [21] identified a genomic variant of HaHV-1 in Taiwanese *H. diversicolor supertexta* suffering from chronic morbidity lasting up to several months and causing up to 80% mortality. PCR and in situ hybridization testing differentiated the virus from the isolate responsible for causing acute mortalities. In addition, this new isolate was found in hemocytes rather than in nervous tissues. The authors concluded that a new pathotype of HaHV-1 had been found.

Another investigation carried out by Bai et al. [10] revealed that the tropism of the Taiwanese HaHV-1 isolate includes both neural tissue and hemocytes; however, no mention was made regarding a pathotype variant. These data suggest that we may be witnessing the appearance of new HaHV-1 variants presenting different pathogenicity and tropism. Full genome sequencing of these isolates and experimental in vivo challenges of different abalone species under different temperature conditions would be required to fully understand the epidemiology of these isolates.

## 3. Diagnostic Tests

Histopathology has been used to diagnose AVG, presumed to be caused by HaHV-1 due to the prominent neurological lesions induced by the virus in abalone species from Taiwan and Australia [6,11]. Additional techniques, such as transmission electron microscopy, can be used to confirm the presence of HaHV-1. However, both techniques are time-consuming and inadequate for epidemiological studies because herpesviruses may persist in clinically healthy hosts (carrier or latent-state infection) and recur under stressful conditions [22]. Therefore, in order to provide rapid, sensitive, and specific diagnosis of HaHV-1, molecular methods were developed.

Chen et al. [23] successfully developed a conventional PCR (cPCR) assay targeting the DNA polymerase gene of HaHV-1. Upon sequencing and analysis, the researchers showed that there is 99% homology in both the nucleotide sequence and amino acid sequence between the Taiwanese and Australian HaHV-1 isolates. In addition, based on viral genome sequence information obtained from the full genome (211,518 base pairs; NCBI Reference Sequence: NC_018874.1) sequence of HaHV-1 Victoria isolate [14], a HaHV-1-specific quantitative PCR (qPCR TaqMan) targeting the ‘open reading frame 49’ (ORF49) was developed for the detection and identification of HaHV-1 [24]. The assay was shown to be specific as it did not detect other viruses from either the *Herpesvirales* or the *Iridovirales* orders, which have genome sequence similarities. In addition, the qPCR ORF49 assay was also able to detect DNA from the Taiwanese HaHV-1 isolate, making the assay a HaHV-1 broad detection diagnostic tool [24]. Because the assay ORF49 did not detect all the HaHV-1 isolates present in Australia, two other TaqMan assays (ORF66 and ORF77) targeting different ORFs of the viral genome have also been developed based on the work from Cowley et al. [13] and validated using samples from hundreds of Australian wild abalone [25]. In order to establish the best assay (ORF49, 66, 77) for detecting HaHV-1 in healthy infected animals, Caraguel et al. [25] used a Bayesian latent class analysis to estimate diagnostic sensitivity, diagnostic specificity, and likelihood ratios of positive and negative test results for each individual test and for all possible combinations of test pairs interpreted either in series or in parallel. They found that assays ORF49 and ORF66 when performed independently, yet interpreted in parallel, performed the best, both analytically and diagnostically. This allows to demonstrate freedom from HaHV-1 in an established population of abalone and to certify individual abalone free from HaHV-1 for trade or movement purposes. Details on the use of these assays as well as a cPCR assay developed by the same team are presented in the OIE Manual of Aquatic Animal Diseases [26].

An additional test, a loop-mediated isothermal amplification procedure (LAMP), was also developed to detect HaHV-1 nucleic acids with high sensitivity, specificity, and rapidity under isothermal conditions [27]. The LAMP is a cheaper alternative test that can be carried out in the field. The authors reported that the LAMP assay enabled the detection of small amounts of HaHV-1 DNA rapidly with high sensitivity and specificity (Table 1). As an additional molecular tool to locate viral replication sites, Corbeil et al. [28] used an in situ hybridization assay that proved useful for diagnostic as well as research purposes, such as visualizing the progression of virus replication in tissues. Gene sequence differences do exist between virus isolates from Taiwan and Australia, suggesting that the use of multiple assays and sequencing of critical gene targets (e.g., helicases) would ensure the detection and identification of current and potential emerging isolates of HaHV-1.

## 4. Experimental Disease Model

In order to characterize as well as understand the virus-host interaction, Corbeil et al. [28] developed a laboratory immersion challenge system composed of 2 L tanks supplied with natural sea water maintained at 16 °C via controlled ambient air temperature. Water aeration was provided using airline tubing and air stones. Abalone were fed commercially available pelleted food for the duration of the experiments. In one instance, by means of a time course study, the researchers established the earliest time points from which molecular detection of the virus is achieved. Results showed that at 36 h post-immersion challenge, the virus was detected by qPCR, while 48 h was the earliest time point for viral detection when using in situ hybridization. In addition, the development of histological lesions in comparison with the onset of clinical signs of disease were observed 60 h post-challenge.

Experimental immersion and injection challenges were also performed to investigate the effects of various physicochemical treatments on HaHV-1 with the aim of developing effective and safe disinfection treatments suitable for land-based aquaculture facilities as well as the wild abalone fisheries sector [29]. To determine the stability of HaHV-1 at different temperatures, the virus was held at 4, 15, and 25 °C for 1, 5, and 12 d prior to the immersion challenge of naïve abalone. Mortality curves indicated that when held for one day in sea water at 4 and 15 °C, the virus remained infectious and highly pathogenic, while longer time frames and a higher temperature compromised the virus viability.

An additional experiment was performed to determine the virucidal efficacy of three disinfectants (calcium hypochlorite, *Buffodine*, and a nonionic surfactant). The disinfectants were used at various doses and timeframes to treat HaHV-1 prior to injection and immersion challenges. Results showed that *Buffodine* and the nonionic surfactant were effective at inactivating the virus with no detectable adverse effects on the abalone’s health [29]. Subsequent trials have been performed by Yumba Aquaculture in Australia to further evaluate the safety and efficacy of these chemicals as well as others in a recirculating aquaculture system in order to be approved by the Australian Pesticide and Veterinary Medicines Authority [personal communication].

Currently, physical cleaning of fomites and tanks and drying between stocking events are the standard procedures used in the Australian aquaculture industry. Specific companies may have additional protocols described in each farm biosecurity plan, a major element of which is prevention of entry of organisms to the farm, and protocols on introduction of disease-free abalone stock. Chemicals are generally not used with the exception of hatchery and nursery tanks, which are cleaned with diluted hypochlorite solutions and freshwater rinsing and drying [personal communication].

## 5. Virus-Host Interaction

With the aim of improving AVG resistance amongst family lines of abalone bred for superior growth and meat texture for the aquaculture abalone industry in Victoria, a study was carried out to evaluate the existence of a genetic basis for resistance to AVG. Corbeil et al. [30] performed experimental transmission trials carried out on 49 family lines of *H. laevigata* grown in an aquaculture facility. Families were evaluated based on the number of days to mortality and viral load at death for each abalone. Data suggested that some abalone families presented differences in susceptibility to AVG; however, the small degree of “resistance”, manifested as a delay to mortality rather than survival to the disease, would not justify undertaking a lengthy and expensive breeding program in order to improve such trait [30].

When AVG spread along the coast of Victoria in Australia, some abalone from the affected populations survived the epizootic and thus may have become naturally resistant to the disease. To test this hypothesis, *H. rubra* from five reefs within the geographical range of the outbreak were collected and tested for resistance to infection and disease using the immersion challenge protocol [31]. Mature survivors and juvenile ‘‘new recruits’’ were exposed to the virus to determine the presence of any potential resistance amongst abalone subpopulations to the virus. Morbidity curves for the wild abalone groups (both mature and juvenile) were similar to those of the susceptible, naïve, farmed hybrid abalone (positive control) groups. Results suggested that the surviving wild abalone were not resistant to AVG and that the survival of some wild abalone during the outbreak starting in 2006 was probably due to the abalone not being exposed to pathogenic doses of the virus. However, some have argued that the small sample size of animals as well as the limited number of sampling sites were a limitation to the study and that AVG-resistant abalone could still be present in the environment. Furthermore, no DNA sequencing was performed on the abalone samples, and therefore a differentiating technique, such as microsatellite analysis, could not be used to compare subpopulations to locate potential mutations responsible for AVG resistance. A current genomic analysis study is currently being carried out by researchers in Australia aiming to remedy this knowledge gap using a broader range for animal sampling as well as a larger number of subpopulations of wild abalone [personal communication].

Subsequent to the AVG outbreak in Victoria, four HaHV-1 isolates were identified in Tasmania in processing plants but did not cause outbreaks in wild abalone populations [12]. The relative pathogenicity of these isolates was evaluated on abalone stocks originating from different states in Australia (Tasmania, Victoria, and South Australia). Results showed that all HaHV-1 variants caused disease and mortality in all the abalone stocks tested (*H. laevigata*, *H. rubra*, and *H. conicopora*) [32], highlighting the vulnerability of the whole abalone fishery in Australia to future AVG outbreaks. Conversely, Corbeil et al. [33] demonstrated that *H. iris*, the New Zealand päua, is highly resistant to disease and infection when challenged by immersion or injection with HaHV-1. This resistance contrasted with the disease susceptibility shown by most other *Haliotis* species tested to date [6,19,32]. The mechanism underlying disease resistance remained elusive, however, a further study at the gene transcriptional level showed that the päua upregulated broad classes of genes that contained chitin-binding peritrophin-A domains [34]. The upregulated chitinases may play a role as immune regulators as this molecule has been found to play a role in animal and plant immunity [35]. The päua also mounted an acute inflammatory response, including the upregulation of VAP-1, an important adhesion molecule for lymphocytes in mammals. Moreover, blood coagulation pathways were broadly dysregulated in the päua, which may have been manipulated by the virus [34].

On a similar line of investigation, a study conducted on the comparative gene expression between the päua and AVG susceptible Australian hybrid abalone (*Haliotis rubra* × *H. laevigata*) [36] showed that the päua and susceptible hybrid abalone have very distinctive early response pathways to viral challenge, with the päua predominantly upregulating transcripts involved in extracellular matrix remodeling. These transcripts were absent in the susceptible Australian abalone.

A similar study by Bai et al. [10] aimed at evaluating AVG resistance in two main cultivated abalone species (*H. diversicolor supertexta* and *H. discus hannai*) in China. The two species were challenged with a HaHV-1 (CN2003) specimen sampled in 2003 in China using three different challenge methods (injection, immersion, and cohabitation). Results showed that *H. diversicolor supertexta* was highly susceptible to HaHV-1 infection and suffered from acute mortalities through all three challenge methods, while *H. discus hannai* was not susceptible to the viral infection. Histopathology combined with transmission electron microscopy and qPCR analysis revealed that the tropism of HaHV-1 (CN2003) includes both neural tissue and hemocytes. The mechanisms of resistance in *H. discus hannai* have not yet been identified.

## 6. Molecular Vaccination/RNAi in Aquatic Invertebrates

Unlike mammals, mollusks do not have an adaptive immune system that provides a long-term protection against pathogens; rather, they possess an innate immune system that relies on cellular responses such as hemocyte phagocytosis and mechanisms of pathogen recognition and activation of nonspecific molecules [37,38,39]. However, studies on RNA interference (RNAi) or RNA silencing, which is a post-transcriptional gene silencing mechanism conserved among eukaryotes as a natural defense mechanism to protect the genome against invasion by mobile genetic elements, such as viruses and transposons [40,41], is a promising field for “vaccination” of invertebrates. RNAi appears to not have been widely used in molluscan species despite their importance as seafood in aquaculture where it could be applied against infectious diseases [42]. To date, most studies regarding innate immunity and RNAi of mollusks have been performed on the Pacific oyster (*Crassostrea gigas*) [38,43,44] using poly(I:C) as viral mimic and demonstrated similarity in responses with the vertebrate interferon pathway that limits viral replication in vivo, extending even to multigenerational immune priming, therefore exhibiting a form of innate immune memory.

In addition, Robalino et al. [45] showed that in the prawn *Litopeneaus vannamei*, the induction of an antiviral state is triggered by nonspecific nucleotide sequences, therefore distinct from the traditional/recognized sequence-specific dsRNA-mediated genetic interference phenomenon. These studies have demonstrated that an invertebrate immune system, like its vertebrate counterpart, can recognize double-stranded RNA (dsRNA) as a virus-associated molecular pattern, resulting in the activation of an innate antiviral response.

Other studies focusing on abalone have shown that they do possess the RNA-induced silencing complex for RNAi functions [46] present in more recently evolved eukaryotes. This would suggest that the use of RNAi could possibly be a new tool for developing cost-effective treatments through feed delivery [44,47] against diseases in mollusks, including abalone. Following this reasoning, research is ongoing to evaluate the efficacy of RNAi molecules to inhibit HaHV-1 replication in hybrid abalone challenged by immersion [personal communication]. A successful outcome would open an opportunity to further investigate the potential of the still poorly understood long-lasting/transgenerational immune priming induced through dsRNA exposure [48,49,50,51] and potentially provide the abalone industry with a “molecular vaccine” against AVG.

## 7. Future of Abalone Production

Although yields of wild abalone fisheries worldwide have flattened [17,18] due to habitat degradation, pollution, and overfishing, the land-based farm production of abalone has increased steadily in the last few years and is expected to continue growing in the near future [17]. Improved knowledge in the field of abalone genomics, innate immunity, disease resistance, and molecular vaccination will provide platforms to support researchers, growers, and legislators to keep increasing production in both aquaculture and in wild fisheries well into the future.

## Figures and Tables

**Table 1:** Molecular diagnostic tests, HaHV-1 specific.

Test	Analytical Sensitivity Viral Gene Copies (v.g.c.)	Characteristics
cPCR [23]	2000 v.g.c.	DNA sequencing verifies target sequence and variation.
TaqMan ORF49 [25]	20 v.g.c.	Fast, sensitive, specific.
TaqMan ORF66[25]	20 v.g.c.	Fast, sensitive, specific.
TaqMan ORF77 [25]	20 v.g.c.	Fast, sensitive, specific.
In situ hybridization [26]	Not evaluated	Visualization of DNA in infected tissues.
LAMP [27]	20 v.g.c.	Fast, sensitive, specific, low cost, and field application.
